# Peripheral magnetic theta burst stimulation to muscles can effectively reduce spasticity: a randomized controlled trial

**DOI:** 10.1186/s12984-022-00985-w

**Published:** 2022-01-16

**Authors:** Nevine El Nahas, Fatma Fathalla Kenawy, Eman Hamid Abd Eldayem, Tamer M. Roushdy, Shahinaz M. Helmy, Ahmed Zaki Akl, Aya Ahmed Ashour, Tamer H. Emara, Marwa Mohamed Moawad, Randa M. Amin, Ahmed M. Elbokl

**Affiliations:** 1 Ain Shams Neuromodulation Research Lab, Neurology Department, Faculty of Medicine, Ain Shams University, P. O. Box 1159, Cairo, Egypt; 2 Neurology Specialist, Ministry of Health, Cairo, Egypt

**Keywords:** Spasticity, Theta Burst Stimulation, Peripheral Magnetic Stimulation, Botulinum Toxin Dose

## Abstract

**Background:**

Spasticity is a common complication of many neurological diseases and despite contributing much disability; the available therapeutic options are limited. Peripheral magnetic stimulation is one promising option**.** In this study, we investigated whether peripheral intermittent theta burst stimulation (piTBS) will reduce spasticity when applied directly on spastic muscles.

**Methods:**

In this sham-controlled study, eight successive sessions of piTBS were applied directly to spastic muscles with supra threshold intensity. Assessment was done by modified Ashworth scale (mAS) and estimated Botulinum toxin dose (eBTD) at baseline and after the 8th session in both active and sham groups.

**Results:**

A total of 120 spastic muscles of 36 patients were included in the analysis. Significant reduction of mAS and eBTD was found in the active compared to sham group (p < 0.001). The difference in mAS was also significant when tested in upper limb and lower limb subgroups. The degree of reduction in mAS was positively correlated with the baseline scores in the active group.

**Conclusion:**

piTBS could be a promising method to reduce spasticity and eBTD. It consumes less time than standard high frequency protocols without compromising treatment efficacy.

*Trial registration:* Clinical trial registry number: PACTR202009622405087. Retrospectively Registered 14th September, 2020.

**Supplementary Information:**

The online version contains supplementary material available at 10.1186/s12984-022-00985-w.

## Introduction

Spasticity is a disabling motor disorder which commonly complicates many neurological diseases. It represents one of the components of upper motor neuron syndrome and is characterized clinically by velocity dependent increase in muscle tone and stretch reflex [1]. Its mechanism is related to loss of supraspinal activation of intraspinal inhibitory circuits on both Ia muscle spindle afferents (presynaptic inhibition) and alpha motor neurons (post synaptic inhibition) [2]. Another possible mechanism that is apparently not under supraspinal control and not mediated by the intraspinal inhibitory circuits is the reduction in post activation depression [2]. Post activation depression reflects an intrinsic neuronal ability to decrease neurotransmitter release following repetitive stimulation of Ia afferents [3]. It was found that limb immobilization and its related changes of mechanical muscle properties is an additional cause of spasticity through reduction of post activation depression of Ia muscle spindle afferents, a mechanism that might play a pivotal role in development of spasticity [4, 5]. Moreover, the delayed appearance of spasticity after acute neurological insult suggests underlying abnormal plasticity, occurring in the spinal cord and also in the brain and that spasticity is beyond being just a release phenomenon [2].The prevalence of spasticity is variable in different neurological diseases. For example, leg spasticity is reported in about 41–66% in patients with multiple sclerosis, 28–38% in patients with stroke, and 13% in patients with traumatic brain injury [6]. Thus, spasticity is a common problem that has its negative implications on motor functions, can cause pain and end up in deformities.

Among the conventional treatments available for spasticity, only Botulinum toxin injections have been proven effective in spasticity reduction [7]. However, the limitations of cost, the need for repeated injections and the limited effectiveness in higher grades of spasticity are major concerns in clinical settings that call for other alternatives.

Non-invasive peripheral stimulation is a promising option that could contribute to motor recovery specially the relatively novel repetitive peripheral magnetic stimulation (rPMS), that adopts the same techniques of cranial stimulation, yet applies the coil to the muscle or nerve [8]. rPMS was reported to reduce spasticity in wrist and finger flexors with active but not with sham stimulation [9].

Also, improvement of kinematics of finger movements was observed following rPMS and that was associated with activation of the parieto-premotor network as shown by PET study. This denotes that rPMS has a central modulatory effect on the brain [10].

rPMS is assumed to act by generating massive proprioceptive inflow either directly by stimulating Ia sensory afferents or indirectly by the repetitive muscle/joint contractions induced by magnetic pulses [8]. This proprioceptive inflow would influence and modulate the activity of the neuronal networks involved in motor control. Being painless, with deeper penetration and preferentially recruiting the proprioceptive afferents, rPMS might be more advantageous over the more popular trans-cutaneous electrical stimulation for motor recovery. It can be a treatment option in conditions like spasticity through its ability to produce repetitive muscle contractions [11]. Nevertheless, studies of rPMS effectiveness in spasticity are still scant and diverse. [12–14].

One form of magnetic stimulation is theta burst stimulation which is a patterned form using less pulses and shorter duration of stimulation than typical repetitive transcranial magnetic stimulation paradigms. Intermittent theta burst stimulation (iTBS) is one form of this patterned stimulation that has a stimulatory effect similar to high frequency rTMS [15] and previous studies have shown that it is as effective as standard high frequency rTMS protocol for depression[16]. However, iTBS can be delivered over a period of 3 min as compared to 37.5 min for the standard 10 Hz protocol of high frequency magnetic stimulation. Applying the 10 Hz protocol for several muscles per patient per session seems too lengthy to be practical. Thus, using iTBS to target several spastic muscles for each patient, we can increase the number of treated patients, without compromising the clinical benefit.

Therefore, in this pilot study, we investigated the efficacy of peripheral intermittent theta burst stimulation (piTBS) applied directly on the spastic muscle belly as shown by change in modified Ashworth scale (mAS) and on reduction of estimated dose of Botulinum toxin (eBTD). This modality might be more appropriate when the amount of toxin required exceeds the therapeutic dose, or in cases where transcranial magnetic stimulation is contraindicated. Also, it can be more convenient in developing countries where the cost of repeated Botulinum injection is an issue and is more time saving than the previously studied rPMS.

## Methods

### Study design

This study is a randomized double-blind sham-controlled pilot clinical trial that was conducted after the approval of the Ain Shams University faculty of medicine research ethical committee [number FMASU MD 283/2017] prior to recruitment.

### Participants

A total of 50 patients with limb spasticity secondary to various neurological disorders were recruited from the neurology clinics. Sample size was calculated according to previous study [9]. All patients or their relatives gave a written informed consent to participate in the study.

### Inclusion criteria

Age more than 18 yrs., disease duration > 6 months with persistent spasticity in the affected muscle (≥ 1 + by mAS) and no change in anti-spasticity medications for at least one month prior to recruitment.

### Exclusion criteria

Recent Botulinum toxin injection for limb spasticity (< 4 months), a metal plate along the spastic limbs, patients with pacemakers and pregnant females.

### Protocol

The patients were examined for spasticity by an expert neurologist and the number of spastic muscles was recorded for each patient. The patients were randomly allocated to two groups (active and sham) through simple randomization by sealed envelopes with ratio 2:1. Peripheral stimulation by intermittent theta burst (piTB) was done over individual spastic muscles. For the upper limb, stimulation was done over biceps brachii and wrist/finger flexor group. For the lower limbs, stimulation was done over rectus femoris, hamstrings and gastrocnemius/soleus. Each group received a total of 8 sessions of stimulation, one session every other day. The active group received stimulation by an active TMS coil and the sham group by an optically similar sham coil. The sham coil produces sounds similar to active TMS coil but being shielded, it does not produce therapeutic effects. The rater was blinded to the type of stimulation given.

**Fig. 1:**
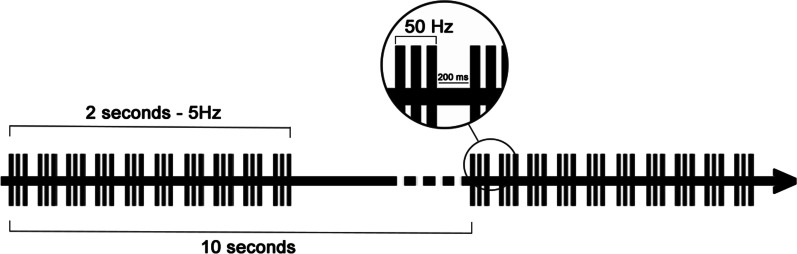
An illustration of intermittent theta burst stimulation: It consists of 10 bursts of 3 pulses at a 50 Hz frequency, lasting for 2 s and repeated at 10 s intervals

The active group received piTBS protocol which consisted of 10 bursts, each of which was composed of three stimuli at 50 Hz, repeated at a theta frequency of 5 Hz every 10 s for a total of 600 stimuli of total duration of 200 s (Fig. 1). Stimulation was administered using a MagVenture dynamic liquid cooled Film Coil (figure of eight, diameter 17 cm, biphasic waveform) connected to a high-frequency magnetic Magpro X100 Stimulator. Stimulation was done with the patient reclining supine and the coil held tangential to the skin with the handle at 90 degrees to the longitudinal axis of the targeted muscle. Stimulus intensity was set at supra-threshold intensity of the stimulated muscle so that visible muscle contraction is perceived by the operator just as a visible muscle flicker. The coil was centered over the muscle belly; on the ventral aspect of lower half of the arm for biceps brachii, the ventral aspect of upper one third of the forearm for the flexors of the wrist and fingers, anterior mid-thigh for rectus femoris, posterior mid-thigh for hamstrings and posterior upper half of leg for gastrocnemius/soleus. Sham group were subjected to the same parameters yet through the use of the sham coil that produces sound only.

### Assessment and outcome measures

Patients were assessed using mAS [17] to measure the degree of spasticity both at baseline and after the 8^th^ session of piTBS. The estimated dose of Onabotulinum toxin A (eBTD) for each muscle was recorded at baseline and after the 8^th^ piTBS session. All examinations were performed by a neurologist expert in neurorehabilitation medicine who was not aware of type of intervention. Also, the eBTD was determined by the same neurologist before and after intervention (however, no BTD was injected over the trial period).

The primary end point was significant reduction of mAS and the secondary end point was significant reduction of the eBTD.

### Statistical analysis

Statistical analyses were done using SPSS (IBM SPSS ver. 20, NY, USA). Level of significance was defined as *P* < 0.05 and results are referred to as means ± standard deviation.

Means of independent samples were compared using independent T-test and Mann Whitney for normally and non-normally distributed data respectively. For related samples, Wilcoxon signed rank test was used for non-normally distributed data.

Chi-squared tests (Fisher’s Exact test or likelihood ratio) were used for categorical data when appropriate. Spearman’s correlation coefficient was used to measure correlation between two non-normally distributed variables. Effect sizes were computed using the methods outlined by *Olejnik & Algina, 2003* [18].

Mean values for Ashworth scale and eBTD were calculated for the total number of muscles studied in each group. Percent reduction of scores of assessment scales was calculated as 100*(T0–1)/T0 where T0 is the baseline value and T1 is the value after piTBS. Minimal clinically important difference (MCID) in Modified Ashworth scale was defined as a reduction of ≥ 1 point [19].

## Results

A total of 42 patients fulfilled the inclusion criteria (27 in the active and 15 in the sham group). During the study there were 6 dropouts (2 in the active and 4 in the sham): 3 patients dropped out due to transportation logistics, 2 due to change in their antispasticity medication during the study period and 1 patient withdrew from the study because he did not experience the reduction of spasticity that he expected. Eventually, 36 patients completed the study (25 in the active and 11 in the sham group). The total number of stimulated spastic muscles was 120; 76 in the active group (38 in the upper limbs and 38 in the lower limbs) and 44 in the sham group (26 in the upper limbs and 18 in the lower limbs) (see Additional file 1: CONSORT flow diagram). No side effects were reported.

At baseline there were no statistically significant differences between the active and sham groups (Table 1).

**Table 1:** Baseline characteristics of active and sham groups

	Active group (n = 25)	Sham group (n = 11)	p-value
Age (years)	47.88 ± 14.8	41.60 ± 14.9	0.266^a^
Gender			
Male	20 (80%)	7 (63.63%)	0.409^b^
Female	5 (20%)	4 (36.36%)	
Disorders			
CVS	17 (68%)	6 (54.55%)	0.344^c^
MS	3 (12%)	1 (9.09%)	
SCI	4 (16%)	1 (9.09%)	
Other	1 (4%)	3 (27.27%)	
Duration (months)	42.74 ± 52.74	64.09 ± 67.07	0.175^d^

	Active (N = 76)	Sham (N = 44)	p-value
Baseline mAS	2.83 ± 0.78	2.8 ± 0.79	0.982^d^
Baseline eBTD	83.14 ± 47.27	85.68 ± 46.15	0.723^d^

*N* number of subjects*,* *N* number of individual muscles,

*CVS* Cerebrovascular Stroke, *MS* Multiple Sclerosis, *SCI* Spinal Cord Injury, *n* number of cases, *mAS* modified Ashworth Scale, *eBTD* estimated Botulinum Toxin Dose

^a^Independent-samples T-Test

^b^Fisher’s Exact Test

^c^Likelihood ratio

^d^Mann Whitney Test

After piTBS, within-group analysis showed significant reduction of mAS scores and eBTD in both groups. However, effect size calculation showed a high relative effect of active piTBS for mAS and eBTD (0.54, 0.53 respectively) compared to a moderate effect for sham stimulation (0.38, 0.39 respectively) (Table 2).

**Table 2:** Modified Ashworth scale scores and estimated Botulinum toxin doses before and after piTBS within active and sham groups

	Before (T0)	After (T1)	*P*-value	Effect size *r*
Active (*n* = 76)				
mAS	2.83 ± 0.78	2.04 ± 0.71	< 0.001*	0.540
eBTD	83.14 ± 47.27	52.07 ± 42.44	< 0.001*	0.530
Sham (*n* = 44)				
mAS	2.80 ± 0.79	2.55 ± 0.83	< 0.001*	0.380
eBTD	85.68 ± 46.15	77.05 ± 44.71	< 0.001*	0.390

*n* = number of individual muscles, mAS = modified Ashworth Scale, eBTD = estimated Botulinum Toxin Dose

**Fig. 2:**
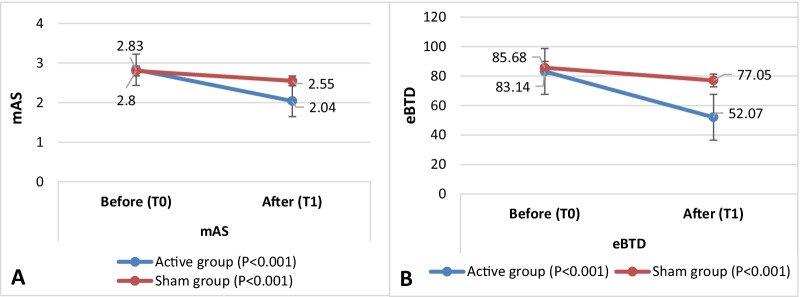
Change in mAS **A** and eBTD **B** across baseline and after piTBS stimulation in active versus sham groups

* Significant p-value (Wilcoxon signed rank test),

Between-group analysis showed significant reduction of spasticity measured by mAS in the active group compared to sham (mean percent reduction in mAS after piTBS: 0.27 ± 0.20; and 0.09 ± 0.15 respectively, P < 0.001). eBTD also showed significant reduction in the active than the sham group (mean percent reduction after piTBS: 0.35 ± 0.34; 0.11 ± 0.15 respectively, P < 0.001). (Fig. 2). MCID for mAS (reduction of ≥ 1 point) was achieved in 41 muscles in the active group compared to 6 in the sham group (P < 0.001) (Table 3).

**Table 3:** Percent reduction in mAS scores and eBTD after piTBS

Total muscles	Active (*n* = 76)	Sham (*n* = 44)	p-value
Percent Reduction in mAS^a^	0.27 ± 0.20	0.09 ± 0.15	< 0.001**
Percent Reduction in eBTD^a^	0.35 ± 0.34	0.11 ± 0.15	< 0.001**
MCID^*^	41	6	< 0.001**

Upper limb	Active (*n* = 38)	Sham (*n* = 26)	p-value
Percent Reduction in mAS^a^	0.26 ± 0.20	0.11 ± 0.17	0.002**
Percent Reduction in eBTD^a^	0.26 ± 0.27	0.13 ± 0.17	0.035**

Lower limb	Active (*n* = 38)	Sham (*n* = 18)	p-value
Percent Reduction in mAS^a^	0.27 ± 0.20	0.07 ± 0.13	< 0.001**
Percent Reduction in eBTD^a^	0.47 ± 0.39	0.08 ± 0.12	< 0.001**

*n* number of individual muscles, *mAS* modified Ashworth Scale, *eBTD* estimated Botulinum Toxin Dose

^a^Percent reduction after piTBS, (Mann Whitney test)

*MCID (minimal clinically important difference): ≥ 1 point reduction on Modified Ashworth scale (Pearson Chi-Square)

**Significant p-value < 0.05

The percent reduction in mAS was also significant when tested in upper limb and lower limb subgroups comparing active to sham (Table 3).

Post-intervention degree of reduction in mAS correlated positively with the baseline scores (rho = 0.268, P = 0.019), that is to say, higher baseline spasticity was associated with more reduction of mAS after intervention.

## Discussion

This sham-controlled study explores the effect of 8 sessions of piTBS applied directly to spastic muscles in the upper and lower limbs secondary to different neurological disorders affecting central nervous system. Post intervention there was a reduction of outcome measures in both active and sham groups, however, the active group had a higher effect size relative to the sham group. Also, comparison of active to sham showed a significantly higher percent reduction of mAS and eBTD. Further analysis showed that upper and lower limb spasticity improved similarly in the active group.

We reviewed the main studies in literature that applied rPMS directly on spastic muscles to treat spasticity and found them to be different in terms of methodology with variable outcomes [12–14, 20]. The main difference between the current study and previous ones lies in the technique of stimulation; while most of these studies used either high or low frequency repetitive magnetic stimulation, we used intermittent theta burst stimulation.

An important observation in similar studies was that the positive effects were more pronounced with higher grades of spasticity than with lower grades. This finding was also observed in our study where higher baseline mAS score correlated with better response. It seems that the amount of baseline spasticity is an important determinant for the response to the rPMS intervention, irrespective of the stimulation protocol. For example, positive effects of rPMS in patients with moderate to severe spasticity (mAS between 3 and 5) were reported by Struppler et al. [12], and more recently by Grozoiu et al. [13], although the former used high frequency rPMS while the latter used low frequency stimulation. In contrast, neither Krewer et al. [14] nor Müller et al. [20] found a relevant effect on muscle tone in patients with mild baseline grades of spasticity, although both studies applied the same high frequency protocol as Struppler [12].

One suggested explanation for the improvement seen with high grade spasticity is that rPMS on muscles possibly works by improving the intrinsic hypertonia (secondary to rheological changes) and the immobility related muscle stiffness rather than improving the reflex spasticity secondary to central mechanisms [20]. Beside the assumed mechanism of massive proprioceptive input through which piTBS might improve spasticity, a direct effect on fibrosis and on the rheological components of chronic hypertonic muscles cannot be excluded especially that these changes are well established in the higher grades of spasticity where rPMS was noticed to be more effective. This possible local effect on hypertonic muscles might also be concluded from the effectiveness of similar local methods of therapy like shock wave therapy that was found to produce long lasting (up to 12 weeks) improvement in spasticity when applied directly on the muscle belly [21].

The ability of piTBS to produce repetitive contraction and relaxation of immobilized muscles mimicking physical exercise might also enhance the mechanism of post activation depression of neurotransmitter release that was found to be reduced in the immobilized limbs. When post activation depression was partially normalized by physical exercise, the hypertonia showed reduction in hemiparetic stroke patients [22].

In the current study, the difference between active and sham stimulation is attributable to improvement of the active group. Contrary to this explanation, Krewer et al. 2014 attributed the significant difference seen in certain muscle groups to deterioration of the corresponding sham group and not actual improvement of the active group. They assumed that rPMS maintained the level of spasticity and prevented its worsening in active cases [14]. However, we argue that in our study, the short period of treatment would not allow for considerable deterioration of spasticity in this group of chronic patients.

As a result of spasticity reduction, we also reported a consequent significant reduction in the eBTD. This might have a great impact in terms of cost burden especially in developing countries where Botulinum toxin is expensive and sometimes difficult to access.

It is worth mentioning that an piTBS session lasts for 3 min while a high frequency 10 Hz magnetic stimulation session lasts for about 40 min. Thus, in most cases of spasticity where several muscles need to be treated, standard high frequency protocols would be time consuming and impractical. On the other hand, botulinum toxin, is known to effectively reduce spasticity in mild and moderate cases but not in severely spastic muscles. piTBS showed good efficacy in high grades of muscle spasticity.

## Conclusion

Repeated sessions of piTBS applied to spastic muscles were effective in decreasing spasticity even in higher grades. This can subsequently lead to reduction of Botulinum toxin dose required for injection. Further studies are recommended to explore the impact of these positive effects on function and long-term effect. If proven to be effective, piTBS would consume less time than standard high frequency protocols without compromising treatment efficacy.

## Limitations

This study was an exploratory one to test the efficacy of piTBS in spasticity, thus a relatively small number of patients was included, larger sample size may be needed to emphasize our findings.

## Supplementary Information

**Additional file 1. **CONSORT flow diagram.

## Data Availability

The datasets generated and/or analyzed during the current study are not yet publicly available due but are available from the corresponding author on reasonable request.

## Abbreviations

mAS: Ashworth scale scores
eBTD: Estimated Botulinum toxin dose
piTBS: Peripheral intermittent theta burst stimulation
rPMS: Repetitive peripheral magnetic stimulation
