# Supplementary material for: Peripheral magnetic theta burst stimulation to muscles can effectively reduce spasticity: a randomized controlled trial
Source: J Neuroeng Rehabil. 2022 Jan 16;19:5. doi: 10.1186/s12984-022-00985-w (PMC8762845; doi:10.1186/s12984-022-00985-w)
Supplement: Supplementary file 1 — Additional file 1. CONSORT flow diagram. [file 12984_2022_985_MOESM1_ESM.doc]

**
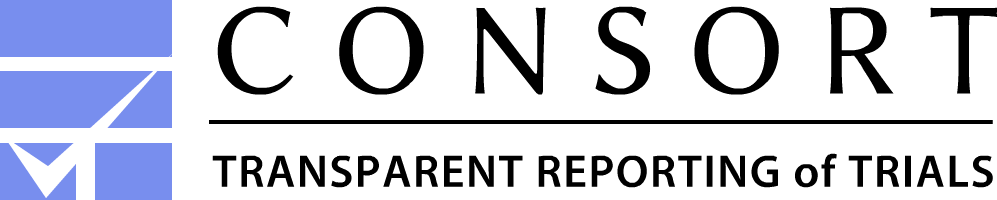
**

**CONSORT 2010 Flow Diagram**

**Allocation**

**Analysis**

**Follow-Up**

**Enrollment**

Assessed for eligibility (n= 50)

Excluded (n= 8)

♦  Not meeting inclusion criteria (n= 7 )

♦  Declined to participate (n= 1 )

♦  Other reasons (n= 0)

Analysed (n= 25 )
♦ Excluded from analysis (give reasons) (n=0 )

Lost to follow-up () (n= 0 )

Discontinued intervention (transportation logisitics) (n=2 )

Allocated to active piTBS (n=27 )

Number of muscles = 76

♦ Received allocated intervention (n=27 )

♦ Did not receive allocated intervention (n= 0 )

Lost to follow-up () (n= 0 )

Discontinued intervention (n=4) (transportation logistics 1), (change of antispasticity medication 2, lack of response 1)

Allocated to sham piTBS (n=15 )

Number of muscles =44

♦ Received allocated intervention (n=15 )

♦ Did not receive allocated intervention (n= 0 )

Analysed (n=11 )
♦ Excluded from analysis (give reasons) (n=0 )

Randomized (n= 42)
